# Women in chemistry: Q&A with Dr Laurie Barge

**DOI:** 10.1038/s42004-025-01442-0

**Published:** 2025-02-22

## Abstract

Dr. Laurie Barge is a Research Scientist in Astrobiology at the NASA Jet Propulsion Laboratory, California Institute of Technology.

Dr. Laurie Barge is a Research Scientist in Astrobiology at the NASA Jet Propulsion Laboratory, California Institute of Technology. Barge co-leads the JPL Origins and Habitability Laboratory which studies how life can emerge and be detected in planetary environments; she is interested in hydrothermal vents as planetary analogs and investigates how prebiotic chemistry can emerge on rocky and ocean worlds. Barge is the HiRISE Investigation Scientist on NASA’s Mars Reconnaissance Orbiter, a Mars Science Laboratory Participating Scientist, and JPL’s Program Area Scientist for Ocean Worlds.

**Figure:**
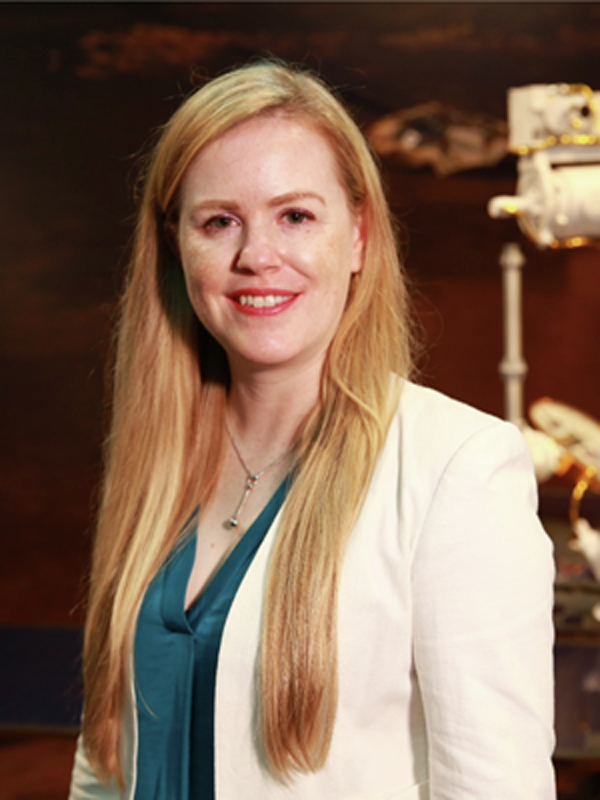
USC/Mike Glier

Why did you choose to be a scientist?

I have always been a space nerd; I was that kid who always read books about the stars and planets; I watched Star Trek; I was into all the sci-fi literature. I always thought I should major in astronomy in college, though honestly, I didn’t think much farther ahead than that until I actually got to college. I majored in astrophysics, and around junior year I discovered the field of astrobiology, which is the study of the origin, evolution, and distribution of life in the universe. This perfectly explained the reasons I was interested in space and planetary science, so I decided to focus my career on astrobiology from then on. After I finished my undergrad degree, I went off to grad school in geoscience – a scary field switch at the time, having never even taken a geology class – but it turned out all the math I learned as an astrophysics major was helpful in geology as well. I first started doing lab work during my PhD, and that’s when I got into chemistry. The lab component ended up being my favorite aspect of research and so I continued doing lab work in my postdoc. Today, I work on several Mars missions and our research group at NASA JPL studies the origin of life and ways to look for life on other planets.

What direction do you think your research field should go in?

My main interest in astrobiology is the origin of life, and in particular, whether life could also emerge on other planets. There have been many origins of life experimental studies, and when they involve geological conditions, it’s usually relevant to the early Earth (which makes sense since this is the planet where we know we have life). But other planets may have had very different environmental conditions than Earth. And even on early Earth, there are lots of questions remaining about what the conditions were in specific types of environments. I think an important direction for the origin of life research is to increase studies of how environmental conditions affect prebiotic reactions. For example, do the relative abundances of organic products change depending on pH or the presence of minerals? How do the presence of geological oxidants or reductants affect organic reaction pathways? Are there geological species (e.g. inorganic ions or metals) that affect prebiotic reactions even though they are not technically part of the reaction, for example, by changing the redox state of another component that does react with an organic precursor? These types of studies could expand our knowledge of the origin of life on Earth and could be crucial for understanding to what extent an Earth-like origin of life might be able to happen on other planets like Mars or ocean worlds.

What scientific development are you currently most excited about?

I’m excited about the discoveries from planetary spaceflight missions regarding organics on other worlds. For example on Saturn’s moon Enceladus, organics as well as phosphate (thought to be a necessary ingredient for habitability) were found in its plume material that has vented out into space from the subsurface ocean. We don’t yet know the source of the organics, but it’s possible they could be produced by water/rock reactions inside Enceladus – similar to how organics could be produced in deep sea vents on Earth. So an exciting question is, how similar could this organic chemistry inside Enceladus be to the chemistry that gave rise to life on Earth? Organics have also been found on Mars, and organic chemistry could have occurred on ancient Mars in the past but again, it’s not known if that ever gave rise to life. But even if there is no life on these worlds, what if we could be witnessing signs of prebiotic chemistry happening today or in the past? That would be huge since the origin of life chemistry is the one thing we can not find in Earth field sites – billions of years of a biosphere have suppressed that abiotic chemistry, so the only place we can really study the origin of life chemistry is in the lab. Unless of course, prebiotic reactions could be happening on other planets, in which case future missions might be able to inform us about how life can get started.

What do you most (and least) enjoy about being a scientific researcher?

The part of being a scientist I enjoy most is mentoring my students and postdocs, and working with my research group to make discoveries together. It’s a pleasure to have students and postdocs since they can focus deeply on aspects of the group’s bigger science vision and accomplish things I would not be able to do by myself. As a grad student and postdoc, I enjoyed my research, but I had to be very focused on one or at most several topics, and even though I was getting all these other research ideas I had to put them aside to finish those early career stages. But now as a PI I get to bring those ideas back and develop them with my own grad students as part of their theses, or have postdocs spin off new ideas of their own. Even the proposal writing aspect, which I know is not everyone’s favorite, I do enjoy it since I find it interesting to do these thought experiments of how I would carry out a new study (I actually find the proposal process quite helpful in terms of planning).

I would say the part of research I like the least is that it can be overwhelming since the job of being a scientist is actually many jobs in one. You must at the same time be a mentor, a group manager, analyze data, write papers, write proposals, manage a lab and instruments, do media and outreach, deal with budgets and accounting, serve on committees at the institute or agency, be a public speaker, etc. Individually a lot of these are interesting and fun types of work, but all together it can be quite overwhelming, especially in the early career where you have to get competent at everything all at once. Even now, every time I think I have one piece going great, I realize there’s another piece that I’ve neglected, and there’s just not enough time in the day to do it all. I think one has to really invest in creating systems for organization and delegation to be successful and have a good work-life balance as a scientist.

Has geographical location or specific institute membership played a role in your experience as a woman in chemistry?

Yes I would say so. I’ve been a scientist in the Los Angeles area for over 20 years now. Being in such a big metro area definitely has its advantages since there are so many scientific institutes here – including all sorts of universities, companies and nonprofits and museums, and of course, NASA JPL where I now work. No matter what stage I was at in my career, there was a wide social network of other scientists and institutes if I wanted to tap into that, and I had a big support network that wasn’t dependent on my specific institute. For example, I got to know other chemists through my professional society’s regional chapter; I befriended folks at a nonprofit in the area and we worked together on some projects; I got to know professors at the community colleges and collaborate with them on different initiatives. In lab sciences, this kind of in-person local network is beneficial and provides one with a useful support system since all kinds of things can happen in the lab. For example, one time our −80 °C freezer was down but we had an important sample arriving; and a local collaborator at another institute received it and stored it for us. (Much of this isn’t particular to being a woman in science, but also, the large amount of professionals in my field in this region does make it easier to connect with other women in chemistry.)

How can individual scientists support and celebrate their women colleagues?

It is great when individual colleagues directly ask what we need and how they can help and really listen to my response. For me, what has helped the most is when colleagues make effort to involve me and other women scientists in things – for example, considering us as speakers, Co-PI’s on proposals, including in those initial discussions when new collaborations are being made, including us in social and networking aspects (dinners at conferences, informal gatherings, lunch/coffee). Much of this is things that individuals may already be doing themselves, and so just making effort to involve women colleagues is an important step that can have quite an impact. There are many other things, of course, that individuals can do, but this is one that comes to mind.

*This interview was conducted by the editors of Communications Chemistry*.

